# A multi-center, randomized, double-blind, placebo-parallel controlled trial for the efficacy and safety of shenfuqiangxin pills in the treatment of chronic heart failure (Heart-Kidney yang deficiency syndrome)

**DOI:** 10.1097/MD.0000000000020271

**Published:** 2020-05-22

**Authors:** Lijun Guo, Hui Yuan, Dawu Zhang, Jian Zhang, Qi Hua, Xiaochang Ma, Keji Chen

**Affiliations:** a National Priority R & D Programmes (2018YFC1707410-02), Cardiovascular Disease Center, Xiyuan Hospital of China Academy of Chinese Medical Sciences; b National Priority R & D Programmes (2018YFC1707410-02), Xiyuan Clinical Medical College of Beijing University of Chinese Medicine; c National priority R & D programmes (2018YFC1707410-02), Fuwai Hospital of Chinese Academy of Medical Sciences; d National Priority R & D Programmes (2018YFC1707410-02), Xuanwu Hospital Capital Medical University, Beijing, China.

**Keywords:** chronic heart failure, protocol, shenfu qiangxin pills, traditional Chinese medicine

## Abstract

**Background:**

Heart failure (HF) is the final stage of various cardiac diseases with poor prognosis. The integrated traditional Chinese medicine (TCM) and western medicine therapy has been considered as a prospective therapeutic strategy for chronic heart failure (CHF). There have been small clinical trials and experimental studies to demonstrate the efficacy of Shenfu Qiangxin Pills (SFQX) for treating CHF, however, there is still a lack of further high-quality trial. This paper describes the protocol for the clinical assessment of SFQX in CHF (heart-kidney Yang deficiency syndrome) patients.

**Methods:**

A randomized, double-blind, parallel-group, placebo-controlled, multi-center trial will assess the efficacy and safety of SFQX in the treatment of CHF. 352 patients with CHF (heart-kidney Yang deficiency syndrome) from 22 hospitals in China will be enrolled. Besides their standardized western medicine, patients will be randomized to receive treatment of either SFQX or placebo for 12 weeks. The primary outcome is the plasma N-terminal pro-B-type natriuretic peptide levels, which will be measured uniformly by the central laboratory. The secondary outcomes include composite endpoint events (hospitalization due to worsening HF, all-cause mortality, other serious cardiovascular events), echocardiography indicators, grades of the New York Heart Association (NYHA) functional classification, the 6-minute walk test (6MWT) results, Minnesota Living With Heart Failure Questionnaire and TCM syndrome scores.

**Discussion:**

The integrated TCM and western medicine therapy has developed into a treatment model in China. The rigorous design of the trial will assure an objective and scientific assessment of the efficacy and safety of SFQX in the treatment of CHF.

**Trial registration:**

Chinese Clinical Trial Registry: ChiCTR2000028777 (registered on January 3, 2020).

## 1 Introduction

Heart failure (HF), as the final stage of various cardiac diseases, is an abnormality of cardiac structure or function.^[1]^ Evidence from China Heart Failure (China-HF) Registry has demonstrated that in-hospital mortality is 4.1% ± 0.3%.^[2]^ Over the last 3 decades, amelioration of treatments and implementation have improved survival and reduced the hospitalization rate in patients with heart failure with reduced ejection fraction (HFrEF).^[3]^ According to the guidelines for chronic heart failure (CHF) treatment, angiotensin receptor neprilysin inhibitor, angiotensin-converting enzyme inhibitors or angiotensin receptor blockers, beta-blockers, aldosterone receptor antagonists, diuretics, digitalis, and vasodilating agents are standard treatments for heart failure.^[4]^ However, the outcomes often remain unsatisfactory. Traditional Chinese medicine (TCM) has the characteristics of multi-target, multi-function and multi-pathway in the process of prevention and treatment of HF. The integrative treatment of western medicine and TCM for HF is associated with increased quality of life, low re-admission rate, and favorable prognosis.^[5]^

China guidelines for the diagnosis and management of heart failure published in 2014 and 2018 all cited evidence from clinical studies of TCM, so more attention has been paid to TCM and it has been widely used in clinical practice. Shenfu qiangxin pills (SFQX, produced by Tianjin Zhongxin Pharmaceutical Group Corporation LTD. Darentang Pharmaceutical Factory), a patent drug made of Ginseng(Renshen), Radix aconiti carmichaeli (Fuzi), rhubarb (Dahuang), the root bark of white mulberry (Sangbaipi), Polyporus umbellata, Semen lepidii (Tinglizi), grifola (Zhuling), have the effects of nourishing Qi, warming Yang, strengthening heart, and promoting diuresis. The pills have been commonly used in TCM for the integrative treatment of patients with CHF who are also diagnosed with heart-kidney Yang deficiency syndrome characterized with palpitations, breathlessness, chest tightness, puffy face and swollen limbs. Experimental researches have shown in animal models that SFQX could reduce water-sodium retention, correct electrolyte disturbance, protect renal and cardiac function via attenuating autophagy and apoptosis.^[6–8]^ Some small-scale clinical studies indicated that SFQX had the advantages of enhancing cardiac contractility and improving heart function.^[9,10]^

However, the explicit role of SFQX in preventing and treating cardiovascular disease remains unclear due to a lack of sound scientific evidence. Currently available randomized controlled trials on SFQX are flawed, with small sample sizes, making it difficult to draw definite conclusions on the actual benefits and harms of it.^[11]^ Hence, further rigorously designed randomized controlled trials are warranted to assess the effect of SFQX in patients with CHF so as to provide high-quality evidence for clinical practice.

We would like to test the hypothesis that patients with CHF will benefit from SFQX and evaluate its safety through a high-quality clinical trial.

## 2 Methods

### 2.1 Objectives

The main objective of this study is to evaluate the safety and efficacy of SFQX so as to provide high-quality research evidence for its clinical practice compared to placebo for treating CHF.

### 2.2 Study design and setting

**Figure 1:**
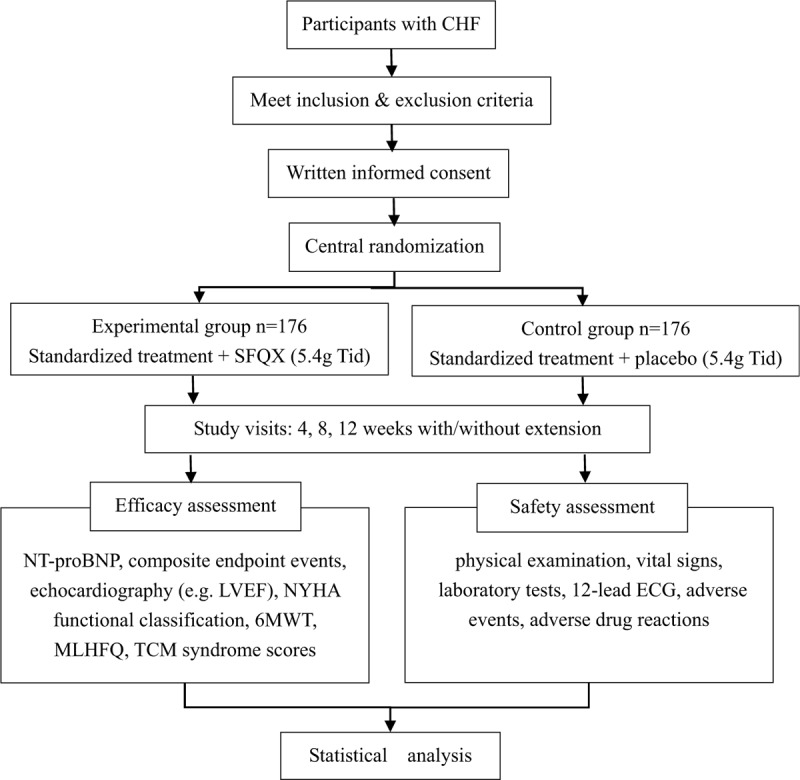
Flowchart of the study. 6MWT = 6-minute walk test, CHF = chronic heart failure, ECG = electrocardiogram, LVEF = left ventricular ejection fraction, MLHFQ = Minnesota Living With Heart Failure questionnaire, NT-proBNP = N-terminal pro-B-type natriuretic peptide, NYHA = New York Heart Association, SFQX = Shenfu Qiangxin pills, TCM = traditional Chinese medicine.

This study is designed as a randomized, double-blind, parallel-group, placebo-controlled, multi-center trial. There are 22 institution hospitals located in different areas of China in this trial. A total of 352 stable patients with CHF, who fulfill the inclusion and exclusion criteria, will be randomized into either an experimental or control group in the ratio of 1:1. The general design of this study is demonstrated in Figure 1.

### 2.3 Participants

#### 2.3.1 Recruitment

Recruitment of the participants began in January 2020 and is expected to be finished in December 2021. Recruitment may be extended depending on registration completion. All participants will be given an informed consent form with a detailed explanation before enrollment, and notified that they may be withdrawn from the trial at any point without penalty. Participants can be recruited directly or openly during the clinical practice or through screening the database.

#### 2.3.2 Diagnostic criteria

(1) Western medicine diagnostic criteria: refer to the 2018 guidelines for the diagnosis and treatment of heart failure in China.^[1]^
(2) TCM syndrome diagnostic criteria : refer to the 2014 expert consensus on diagnosis and treatment of TCM in CHF and 2016 expert consensus on diagnosis and treatment of integrated traditional and western medicine in CHF.^[5,12]^

#### 2.3.3 Inclusion criteria

(1) Participants who meet the diagnostic criteria of CHF, and have a history of CHF for more than 3 months or clinical symptoms of HF for more than 3 months.
(2) Participants with heart-kidney Yang deficiency syndrome based on differentiation of syndromes and treatment.
(3) Aged 18 to 80 years.
(4) Left ventricular ejection fraction (LVEF) < 40%.
(5) New York Heart Association (NYHA) class II to IV.
(6) N-terminal pro-B-type natriuretic peptide (NT-proBNP) ≥450 pg/ml.
(7) Participants who have received standardized western medicine treatment with the optimal therapeutic dose for at least 2 weeks.
(8) Participants who have not used TCM for heart failure within 2 weeks before enrollment.
(9) Submitted informed consent.

##### 2.3.3.1 Exclusion criteria

(1) Participants who have undergone coronary revascularization and cardiac resynchronization therapy within 12 weeks or cardiac resynchronization therapy planned within 12 weeks.
(2) Participants with severe primary diseases such as liver and kidney hematopoiesis and so on; alanine transaminase, aspartate transaminase, alkaline phosphatase, and/or serum creatinine values that are 2 times higher than the normal upper limit; serum potassium content >5.5 mmol/L; patients with tumor, severe neuroendocrine diseases and mental illnesses, etc.
(3) Participants with left ventricular outflow tract obstruction, acute myocarditis, hypertrophic cardiomyopathy, restricted cardiomyopathy, aortic aneurysm, arterial dissection, congenital heart disease, severe arrhythmia, and significant hemodynamic changes in unrepaired heart valve disease
(4) Participants with uncontrolled hypertension, systolic blood pressure ≥180 mmHg and/or diastolic blood pressure ≥100 mmHg.
(5) Participants with severe peripheral arterial disease, acute attack of chronic obstructive pulmonary disease, pulmonary vascular disease such as primary pulmonary hypertension or pulmonary hypertension due to autoimmune disease.
(6) Participants who are pregnant or preparing for pregnancy and lactation.
(7) Participants with an allergic constitution, known sensitivity to the study drugs or their ingredients.
(8) Participants in other clinical trials within 1 months.

### 2.4 Study procedure

**Figure 2:**
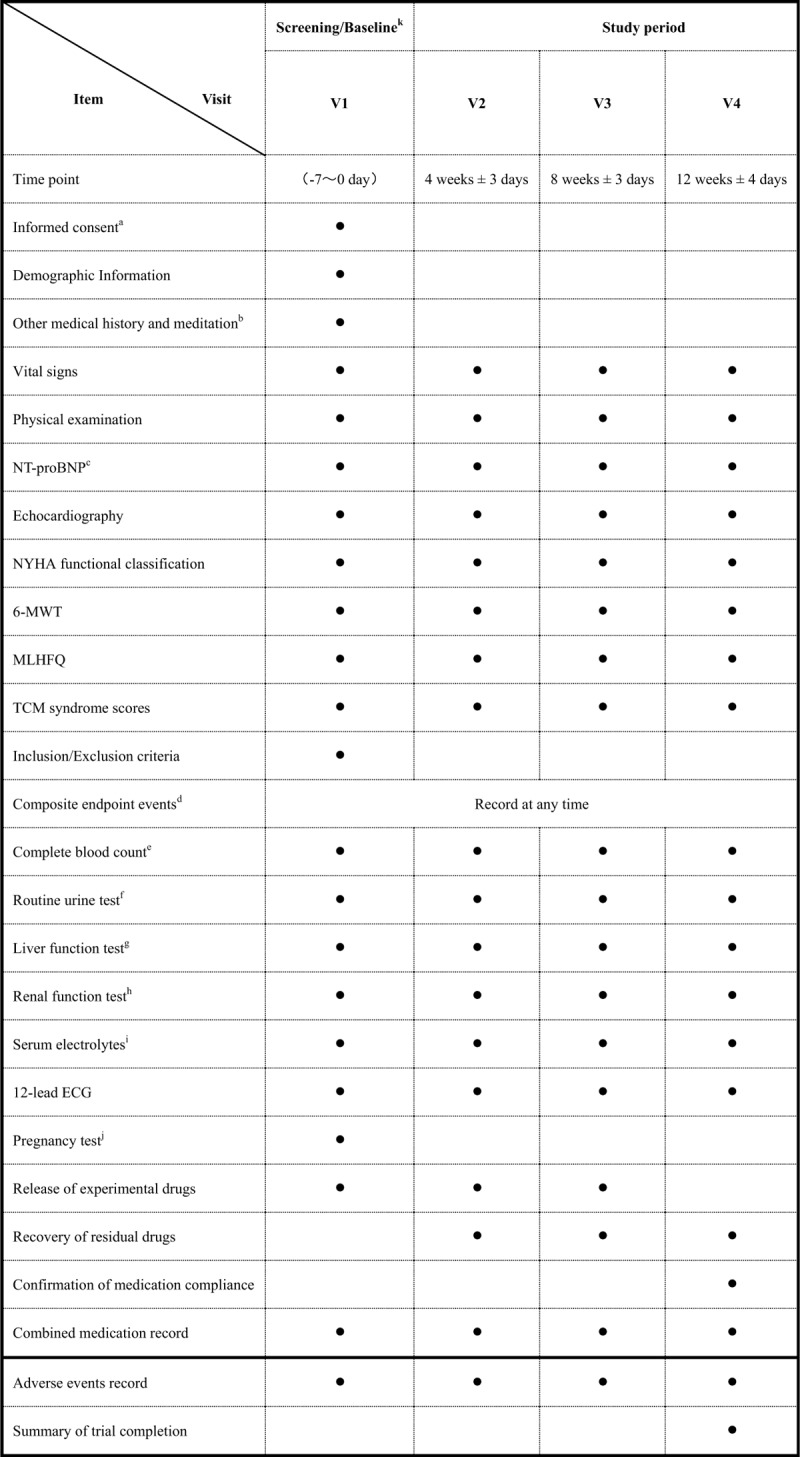
Study visits. A. All tests related to subjects should be carried out after signing the informed consent. B. Patients’ current chronic and acute diseases (referred to as concomitant diseases) will be recorded, along with any history information (referred to as medical history) that the researchers consider meaningful for chronic heart failure and this study. C. At the time of enrollment, the test result of NT-proBNP is based on the laboratory test standard of the sub-central research institutions. Meanwhile, spare samples will be collected and sent to the central laboratory in Beijing for measurement; the statistical results during the visit period are based on the test standard of the central laboratory. D. Composite endpoint events will be recorded at any time, including hospitalization due to worsening heart failure, all-cause mortality, and other serious cardiovascular events. E. Complete blood count includes white blood cell count, red blood cell count, hemoglobin, platelet. F. Routine urine test includes urine leukocytes, urine protein, urine latent blood, urine glucose. G. Liver function test includes alanine aminotransferase, aspartate transaminase, total bilirubin, r-glutamyl transferase, alkaline phosphatase. H. Renal function test includes serum creatinine, blood urea nitrogen. I. Electrolytes include serum potassium, serum natrium, serum chlorine. J. During the screening period, women of childbearing age can be included in the group only when the pregnancy test result is negative; during the study period, contraceptive measures are adopted voluntarily. K. The test results within 7 days before enrollment can be used as baseline data.

The participants who provide written informed consent and meet the inclusion and exclusion criteria will be selected at the screening visit, namely visit 1. Eligible participants will be informed via telephone call and will be asked to visit the trial center within 1 week (visit 1). At visit 1, the investigator will record demographic information, other medical history and medication, vital signs, physical examination and so on, and subjects will be randomized to the experimental or control group. The dosage used in this study is 5.4 g (2 bags) of SFQX or placebo 3 times daily for 12 weeks. Tests and assessments will be performed according to the following schedule: Visit 2 (NT-proBNP, echocardiography, NYHA functional classification, 6MWT, Minnesota Living With Heart Failure questionnaire (MLHFQ), TCM syndrome scores, composite endpoint events, complete blood count, routine urine test, liver function test, renal function test, serum electrolytes, 12-lead electrocardiogram); Visit 3 and Visit4 are the same as above. Detailed assessment schedules are outlined in Figure 2.

### 2.5 Intervention

The shape and appearance of SFQX and placebo are the same: tan herbal pills. Each bag of pills is manufactured at a dosage of 2.7 g, with shelf-life of 18 months. The manufacturing company (Tianjin Zhongxin Pharmaceutical Group Corporation LTD. Darentang Pharmaceutical Factory) follows the regulations for good clinical practice, and controls the quality of the products using their own standards and testing methods.

The principle investigator will receive the products used for this trial from the company and supply them to the drug administrator. The drugs for each subject are in a box consisting of three packages (1 package for each visit period) which are labelled. A package, which includes 168 bags for 28 days plus 18 bags for spare will be prepared at 4-week intervals from visit 1 to 3. All products used in the trial will be recorded, including the amount of the medication, the date of the delivery, and the date of return.

After randomization, the participants will begin taking 5.4 g (2 bags) of SFQX or placebo orally combined with standardized western medicine treatment 3 times daily after meals for 12 weeks.

### 2.6 Randomization, blinding

The central random method is used in this trial where IWRS system is used for central randomization and the assignment scheme is hidden. A block dynamic randomization method is used for randomized allocation. Randomized code generation and drug blinding will be implemented independent of the data. The trial is double-blind where the placebo is the same as SFQX in shape, color, smell, properties, and so on. Both the participants and the investigators will be blinded until completion of the trial.

### 2.7 Sample size calculation

The sample size calculation is based on the proportion of patients with a decrease of NT-proBNP level by at least 30%. According to the reference, 47.95% of the patients in the qili qiangxin capsule group had reductions in NT-proBNP levels by at least 30%, compared with 31.98% of patients in the placebo group.^[13]^ We assume the proportion of patients demonstrating a decrease in NT-proBNP level by at least 30% in the treatment group would be 48%. Therefore, given a type I error rate of α = 0.05, a power of 80% (type II error rate of β = 0.20), the sample size of each group is 146 cases calculated by PASS11 software. Moreover, considering a dropout rate of approximately 20% for randomized patients, a total of 352 patients (experimental group = 176, control group = 176) is required to be randomized for the efficacy analysis.

### 2.8 Statistical analysis

The statistical significance of this study is conducted by 2-sided test and *P* < .05 is considered statistically significant. Statistics software SAS 9.4 will be used for the data analysis. Measurement data are analyzed by paired *t* test, variance analysis, rank sum test, and so on. Enumeration data are analyzed by chi-square test, Fisher exact test, etc. Ranked data are analyzed by Ridit or Cochran-Mantel-Haensel. The analysis data set will be selected from full analysis set, per-protocol set and safety analysis set. Considering the influence of baseline value, covariance analysis or logistic regression analysis will be used to deduct the baseline factor.

### 2.9 Efficacy assessment

#### 2.9.1 Primary outcome

The primary outcome is NT-proBNP, Efficacy assessment of primary outcome is based on either the change in NT-proBNP level or the proportion of the patients with NT-proBNP level decreased by at least 30% from baseline to 12 weeks.

#### 2.9.2 Secondary outcomes

The secondary outcomes will include composite endpoint events (CCEs), echocardiography indicators (LVEF, left ventricular end diastolic diameter, left ventricular end systolic diameter, A/E), grades of NYHA functional classification, 6MWT results, MLHFQ, TCM syndrome scores. Efficacy assessment of secondary outcomes is based on the change in echocardiography indicators, grades of NYHA functional classification, 6MWT results, MLHFQ, TCM syndrome scores from baseline to 12 weeks and the proportion of patients with composite endpoint events at the end of the trial.

### 2.10 Safety assessment and adverse events report

The safety assessment is based on spontaneous reports of adverse events, vital signs, laboratory tests. Vital signs include temperature, blood pressure and heart rate and breathing. Laboratory tests include complete blood count (white blood cell count, red blood cell count, hemoglobin, platelet), routine urine test (urine leukocytes, urine protein, urine latent blood, urine glucose), liver function test (alanine aminotransferase, aspartate transaminase, total bilirubin, r-glutamyl transferase, alkaline phosphatase), renal function test (serum creatinine, blood urea nitrogen), serum electrolytes (serum potassium, serum natrium , serum chlorine ) and 12-lead electrocardiogram.

Adverse events will be recorded at any time during the trial. If any serious adverse event occurs during the trial, the investigator shall immediately take appropriate treatment for the subject, and report to the medical ethics committee of the primary research institution and the sponsor.

### 2.11 Data management

Data collection and management consists of 2 parts: one is Case Record Form (CRF) and another is ResMan public platform in Electronic Data Capture (EDC). Medical information obtained from this trial will be recorded in each patient's CRF and remain confidential which will be checked with the original medical records of the subjects. The researcher's EDC user name and password are specially assigned to enter the data. With the data of each center institution completed and all questions resolved, the project manager and the main researcher need to review the contents of each case again. The data administrator makes a database lock list and saves data management related documents as required. All research data will be kept for 5 years which include confirmation of all subjects, original written informed consent, CRFs, detailed records of drug distribution, etc.

### 2.12 Monitoring

A specialized monitor will be responsible for supervising the entire trial process, regularly review and verify whether the trial is conducted and documented on the basis of plan, standard work guidelines, and relevant regulations.

### 2.13 Ethics and dissemination

This trial protocol was approved by the Ethics Committee of Xiyuan Hospital of China Academy of Chinese Medical Sciences in 12 December 2019 (2019XLA062-4). Any protocol deviations will be approved by the Ethics Committee of Xiyuan Hospital of China Academy of Chinese Medical Sciences. The trial has been registered with the Chinese Clinical Trial Registry. All participants or authorized surrogates will be given a detailed explanation on this trial with the informed consent form and appropriate time to determine consent or assent.

## 3 Discussion

HFrEF is characterized by defects in cardiac contraction. Although the conventional therapeutic approaches in HFrEF management have improved survival rates in patients, the prognosis still remains poor. The efficacy of integrative treatment for CHF with TCM and western medicine has been gradually accredited and the integrative treatment has been considered as an alternative therapeutic strategy for CHF with fewer side effects.^[14]^

As a post-marketed medicine, the composition of SFQX is clear and its quality is assured. Some clinical reports^[15–20]^ have indicated that western medicine combined with SFQX could further improve cardiac function, TCM syndrome, the quality of life and show a clinical curative effect, improve LVEF and narrow left ventricular end diastolic diameter. They may work by reducing plasma brain natriuretic peptide and atrial natriuretic peptide, anti-inflammatory, inhibiting oxidative stress, protecting vascular endothelial function, suppressing rein-angiotensin-aldosterone system system, reversing ventricular remodeling to inhibit myocardial fibrosis. However, there is still a demand on a high-quality and larger-scale clinical research to prove its efficacy and safety in the treatment of CHF.

The study design of this trial has 3 main points.

1. To assess the results of the treatment effectively, a standardized outcome measure is required. We select NT-proBNP as a primary outcome. Plasma will be collected in various hospitals and transported to the central laboratory (the Department of Laboratory Medicine, Xiyuan Hospital of China Academy of Chinese Medical Sciences, Beijing, China) by cold chain for NT-proBNP detection. Plasma NT-proBNP levels were measured by dedicated kit-based NT-proBNP assays (Roche Diagnostics).
2. CHF patients with heart-kidney yang deficiency syndrome will be included on the basis of syndrome differentiation and treatment, which reflects both the advantages and characteristics of TCM and the efficacy evaluation of western medicine. Patients with syndrome characterized by palpitations, shortness of breath or wheezing, fear of cold could be diagnosed.
3. The study protocol was designed with reference to international clinical trial principles. Three-level quality control measures were taken and data were monitored by EDC system.

Quality control supervisors will ensure that investigators adhere to the study protocol in each center institution. We used the random method of dynamic randomization (multiple blinding) of sub-center group to compete for the group, so as to manage data and reduce deviation in the research. The rigorous design of the trial will ensure an objective and scientific assessment of the efficacy and safety of SFQX in the treatment of CHF.

## Acknowledgments

We also thank the following coordination hospitals and directors:

(1) Xiyuan Hospital of China Academy of Chinese Medical Sciences, Xiaochang Ma
(2) Fuwai Hospital, Chinese Academy of Medical Sciences, Jian Zhang/Mei Zhai
(3) Xuanwu Hospital Capital Medical University, Qi Hua
(4) First Teaching Hospital of Tianjin University of TCM, Jingyuan Mao
(5) Second Affiliated Hospital of Tianjin University of TCM, Yingqiang Zhao
(6) Shuguang Hospital Attached to Shanghai TCM University, Xiaolong Wang
(7) The First Affiliated Hospital to Changchun University of Chinese Medicine, Yue Deng
(8) Shengjing Hospital of China Medical University, Zhijun Sun
(9) Xinjiang Uygur Autonomous Region Hospital of Traditional Chinese Medicine, Xiaofeng Wang
(10) Tianjin Chest Hospital, Shutao Chen
(11) Guangdong Hospital of TCM, Weihui Lv
(12) Affiliated Hospital of Jiangxi University of TCM, Zhongyong Liu
(13) Affiliated Zhongshan Hospital of Dalian University, Qin Yu
(14) Shijiazhuang No.1 Hospital, Xitian Hu
(15) Handan First Hospital, Xianzhong Wang
(16) Wuxi Traditional Chinese Medicine Hospital, Shu Lu
(17) Huangshi central hospital, Daoqun Jin
(18) Luoyang Hospital of TCM, Yanling Sun
(19) TCM-integrated Hospital of Southern Medical University, Yiye Zhao
(20) Yuncheng Central Hospital, Xia Wang
(21) Xingtai People's Hospital, Hebei Medical University Affiliated Hospital, Limei Yao
(22) Taiyuan Iron and Steel (Group) Co., Ltd., General Hospital, Qing Ji

The hospitals all above are listed irrespective of ranking.

## Author contributions

**Conceptualization:** Lijun Guo, Dawu Zhang, Xiaochang Ma.

**Supervision:** Xiaochang Ma, Jian Zhang, Qi Hua, Keji Chen

**Writing – original draft:** Lijun Guo, Hui Yuan.

**Writing – review & editing:** Lijun Guo, Hui Yuan, Xiaochang Ma.
